# Helps to Success in Practice

**Published:** 1902-01

**Authors:** George L. Parmele

**Affiliations:** ’70, Hartford, Conn.


### HELPS TO SUCCESS IN PRACTICE.[1]

BY GEORGE L. PARMELE, M.D., D.M.D., '70, HARTFORD, CONN.

At first thought it would hardly seem possible that genealogical information could have been one of the first things to help us in dentistry, or rather to help me into dentistry. But it did. In the days which some of you remember, you veterans of '69 and '70, when the Medical Museum was at the east end of the second story of this old Medical School building, it was the custom at examination-time for the various professors to seat themselves at little tables, arranged around this large room. On the opposite side of the table was a chair for the victim. At a signal given at regular intervals,—every ten minutes, if I remember correctly,—each candidate would move promptly to the next table on the right, like a progressive whist party. In swinging round the circle that day, I came in opposition to Professor Oliver Wendell Holmes, to be roasted upon his anatomical gridiron. Dr. Holmes opened the battle by asking my name, which I answered correctly. "How do you spell your name?" "P-A-R-M-E-L-E." Two correct answers. Dr. Holmes then said, "I had an acquaintance, a fine old gentleman in New York, a dentist, named Eleazar Parmly, but he spelled his name P-a-r-m-l-y." I explained with as much circumlocution as possible the various spellings of the name, and stated that, in spite of this variation, the family in America were all descended from

---

[1] Read before the Harvard Dental Alumni Association, June 24, 1901.

John Parmelin, one of the original founders, in 1669, of Guilford, Conn. He then inquired in what way I was related to Dr. Eleazar Parmly.

Whereupon I had the pleasure of informing him that my great-great-great-grandfather was brother to the great-great-grandfather of Dr. Eleazar Parmly. Now all this genealogical chat occupied time and reduced the anatomical questions to the minimum. Thus did genealogical lore prove useful in my dental experience.

Your secretary has been punching me up for the past two years to read a paper before you, and I have managed to evade him, but this year, in spite of protests, he insisted that, inasmuch as thirty years had passed over my head since I graduated, I ought at least to come and show myself, and let some of my old comrades know that I was still upon earth, and this, I assure you, is about all I am going to do.

I will not detain you long (ten minutes, he said, would be enough; yes, more than enough, you say). Neither am I going to offer apologies for the crudity of my remarks, scratched off at the eleventh hour, in the hurry of pressing affairs, out of as well as in my office. In an adjoining room, among those of my classmates, appears my photograph, and, although I give you my class year, 1870, I defy any of you to pick it out. I assure you, however, that I thought I knew a vast amount more then than I do now. I hardly feel that I have attained enough success to write upon the subject assigned me by your secretary, therefore I have taken the liberty to change the title, so that it reads, "Helps to Success in Practice." It is not my purpose to present a series of mechanical devices or a list of favorite remedies that have helped me, but rather to touch lightly upon one or two points that strike me as aids to success, after one has laid the foundation by earning a diploma from this school.

I offer, not so much the things that have helped me, but rather some things I consider essential to every practitioner. I believe that it would be by far the wiser course for all those entering upon a professional career if they would after graduation serve as assistants with some successful practitioner. There they would gain experience, not only in the manipulation necessary to the care of the dental organs, but learn—what is vastly more important to them at the outset—how to deal with people, and to meet the many petty, but important details of daily routine in a successful office.

"The every-day cares and duties which men call drudgery are the weights and counterpoises of the clock of time, giving the pendulum a true vibration, and its hands a regular motion."

"Of all work that produces result, nine-tenths must be drudgery. There is no work, from the highest to the lowest, which can be done well by any man unwilling to make that sacrifice."

"The public judge us by what we appear to be and by what we have already accomplished. We judge ourselves by what we feel capable of doing." "The talent of success is nothing more than doing what you can do well, and doing well whatever you can do without a thought of fame."

It is the attention to petty details that tells the story of success or failure. Many have failed through the want of appreciation of their importance. A tooth is a little thing, a bundle of cells, yet upon its preservation rest the health and beauty of the individual.

Many patients are so alert and nervous that it is difficult to eradicate the impression that they are to be hurt, and they submit to our manipulations with the firm conviction that they will be hurt; but my experience is that kindly, encouraging talk and gentle handling will gain their confidence and allay their dread to such an extent that we can go on with our operations with no trouble whatever. So true is this in the care of children, that our first efforts should be entirely devoted to subduing their fear and gaining their confidence before attempting to accomplish anything else. From that time on they are yours to command.

The saving of teeth is an important matter, and we, as well as our patients, know that teeth cannot be saved without considerable discomfort; but we should remember that we are working upon living, human beings, and we should hardly be justified, after such manipulations in reporting, as surgeons sometimes do, that "the operation was a success, but the patient died." It is our duty during operations for nervous patients to give them all the encouragement that we can, not by soft, insipid speech or by patting their cheeks, or hanging over them as though they were sick pets, as some dentists have been known to do, but by gentleness of manner and speech convincing them that, although we shall operate with firm and steady hand, we will inflict no unnecessary pain. Each movement should accomplish its definite object, and no uncertain scratching around should be indulged in.

Some of the essentials to success that I will enumerate are:

Cleanliness in all things, in your person, in your surroundings, your linen, and appliances. Not only be clean, but, if possible, let the patient realize it.

Be gentle. Cultivate at all times gentleness of touch and bearing in the handling of your patients, but at the same time be confident and firm.

Endeavor to reduce all the pain and discomforts of the dental chair to the minimum.

Be thorough in examinations, and hold a consultation, so to speak, with your patients, instructing them as to the importance of what you propose doing for their comfort.

Be cheerful, and have your surroundings cheerful.

Be sympathetic, but not aggressively so. Try by kindly suggestions to put them at ease, and convince them of your unwillingness to inflict unnecessary pain.

Provide yourself with short entertaining stories; keep yourself informed upon current events, the latest literature, theatrical events, advances in the arts and sciences, so that at all times you can, after learning what interests your patients, keep their minds off of themselves by occasional chats on the subjects. And, withal, cultivate in yourself the importance of the attention to every little detail that can enhance your patient's comfort. It is of as much importance to know when *not* to do a thing as to know how to do it. To succeed, it is of as much importance to know how to handle your people as to handle instruments.

It has been forcibly impressed upon me of late that the average dentist—mind you, I am not speaking of present company—is apt to be a narrow-minded individual, or, as a friend expressed it not long since, "there are more blamed fools among dentists than among any class of men I deal with." Now why, if true, is that so? One of our famous writers has said, "In great cities we learn to look the world in the face. We shake hands with stern realities. We see ourselves in others. We become acquainted with the motley many-sided life of man." This is true in most walks in life: the merchant, the business-man of every calling, and most professional men go out among their fellow-men; their angularities get worn down; they become polished and made liberal by this contact. The world seems broader to them. They think of things other than their calling. How is it with many dentists? Daily from morn to dewy eve they confine themselves in a small room in contact principally with nervous women and children. They talk shop in their office and out of their office; and if at the start they were broad and liberal, can we wonder that the rut they are in tends to make them narrow and bigoted? Dentistry is a useful, honorable calling, but the world does not revolve around it as such men seem to think.

Let us avoid, then, getting into this rut. Let us mix with the world more, and become interested in things outside our dental world, and let us avoid too much talk of shop.

The specialist should know everything of his specialty, and he should know something of all things, even though not immediately therewith connected. He should be a student, and above all things keep pace with his own science, but should inform himself as to all sciences and the world in general.

So must the dentist's life be a continuation of his student life until he gives up the practice of his profession. The improvements in our art, the daily advances in dental science, are now so rapid that whenever any dental specialist ceases to be a student he falls behind, and is not capable of doing that full justice to his patrons which they have a right to expect. No matter what the defect or deformity he is called upon to treat, if he cannot give it the best and most perfect treatment known at the time, he has failed in his duty.

Friends, my purpose in appearing here to-day has been to meet you, to greet you all, to renew my acquaintance with many, to become acquainted with more. These notes for a paper were simply a pretext of your secretary and myself to get me to this reunion, and I am glad we have succeeded. Take these notes as they are offered. We should know each other better, and I trust we may be spared to meet time and time again.

